# Energy Expenditure Compared to Physical Activity Measured by Accelerometry and Self-Report in Adolescents: A Validation Study

**DOI:** 10.1371/journal.pone.0077036

**Published:** 2013-11-04

**Authors:** Pedro C. Hallal, Felipe F. Reichert, Valerie L. Clark, Kelly L. Cordeira, Ana M. B. Menezes, Simon Eaton, Ulf Ekelund, Jonathan C. Wells

**Affiliations:** 1 Postgraduate Program in Epidemiology, Federal University of Pelotas, Pelotas, Brazil; 2 Childhood Nutrition Research Centre, UCL Institute of Child Health, University College London, London, United Kingdom; 3 Department of Surgery, Institute of Child Health, University College London, London, United Kingdom; 4 MRC Epidemiology Unit, Institute of Child Health, University College London, London, United Kingdom; 5 Department of Sport Medicine, Norwegian School of Sport Sciences, Oslo, Norway; NIDDK/NIH, United States of America

## Abstract

**Background:**

Physical inactivity is responsible for 5.3 million deaths annually worldwide. To measure physical activity energy expenditure, the doubly labeled water (DLW) method is the gold standard. However, questionnaires and accelerometry are more widely used. We compared physical activity measured by accelerometer and questionnaire against total (TEE) and physical activity energy expenditure (PAEE) estimated by DLW.

**Methods:**

TEE, PAEE (TEE minus resting energy expenditure) and body composition were measured using the DLW technique in 25 adolescents (16 girls) aged 13 years living in Pelotas, Brazil. Physical activity was assessed using the Actigraph accelerometer and by self-report. Physical activity data from accelerometry and self-report were tested against energy expenditure data derived from the DLW method. Further, tests were done to assess the ability of moderate-to-vigorous intensity physical activity (MVPA) to predict variability in TEE and to what extent adjustment for fat and fat-free mass predicted the variability in TEE.

**Results:**

TEE varied from 1,265 to 4,143 kcal/day. It was positively correlated with physical activity (counts) estimated by accelerometry (rho  = 0.57; p = 0.003) and with minutes per week of physical activity by questionnaire (rho  = 0.41; p = 0.04). An increase of 10 minutes per day in moderate-to-vigorous intensity physical activity (MVPA) relates to an increase in TEE of 141 kcal/day. PAEE was positively correlated with accelerometry (rho  = 0.64; p = 0.007), but not with minutes per week of physical activity estimated by questionnaire (rho  = 0.30; p = 0.15). Physical activity by accelerometry explained 31% of the vssariability in TEE. By incorporating fat and fat-free mass in the model, we were able to explain 58% of the variability in TEE.

**Conclusion:**

Objectively measured physical activity significantly contributes to the explained variance in both TEE and PAEE in Brazilian youth. Independently, body composition also explains variance in TEE, and should ideally be taken into account when using accelerometry to predict energy expenditure values.

## Introduction

The short and long-term benefits of adolescent physical activity for health are well known [1]. To date, the majority of studies has focused on physical inactivity trends in high-income countries and has resulted in literature gap concerning physical inactivity in low- and middle-income countries, where the types of activities practiced differ from those taking place in high-income settings. Further, there is still much debate concerning how best to express and measure physical activity-related variables that has raised questions on the validity of tools used to measure physical activity data. When expressing physical activity variables, it is essential to differentiate between the concepts of energy expenditure and physical activity. Energy expenditure refers to the act of using energy to conduct a variety of physical processes, including maintaining homeostasis, growth, thermogenesis, and practicing physical activity; while physical activity refers to any body movement produced by skeletal muscles leading to energy expenditure [2].

The doubly labeled water (DLW) technique is considered the gold standard for measuring energy expenditure in free-living individuals. However, the use of DLW is relatively expensive and typically is not feasible for large studies. As a consequence, researchers typically rely on estimates of total energy expenditure (TEE) derived from alternative measurement techniques, which indirectly assess energy expenditure by measuring physical activity energy expenditure (PAEE). To assess the validity of these alternative techniques, the energy expenditure calculated from physical activity in large-scale studies is compared to energy expenditure measured by the DLW technique derived from a subsample of the study population [3]–[7].

Although no gold standard methods are available for the measurement of physical activity, accelerometry is assumed to be the most objective technique for recording gross body movement [8]. Previous studies in children have compared physical activity assessed using accelerometry against PAEE and TEE estimated using the DLW technique [3]–[7]. The results of these studies have varied, with two finding no significant association between DLW and physical activity levels [5], [6], and the other three describing positive correlations [3], [4], [7]. According to the literature, associations between accelerometer-derived physical activity and DLW vary according to the type of accelerometer employed [9] and heterogeneity in the population, including its activity type and level [9], [10], age, and sex [11].

Comparisons of physical activity levels assessed through questionnaires and DLW have also produced heterogeneous findings. A review of 20 validation studies on this topic concluded that the validity of physical activity questionnaires in adults to estimate PAEE is ‘unclear’ [12]. Corder et al. conducted a validation study of four different self-report questionnaires for children and adolescents against DLW and accelerometry and found that that there was no single physical activity questionnaire able accurately to assess all dimensions of PAEE, and that the ability to predict PAEE differed according to the questionnaire used and the age group studied [13].

To test the validity of physical activity measurement tools in Brazilian adolescents, we compared TEE and PAEE estimated by DLW against physical activity assessed by both accelerometry and questionnaires in a sample of adolescents. We further investigated to what extent moderate-to-vigorous intensity physical activity (MVPA) predicted the variability in TEE, and to what extent adjustment for fat and fat-free mass, which might remove the confounding effect of sex [14], predicted the variability in TEE.

## Methods

### Ethics Statement

Human subject considerations were taken throughout all phases of this study and written informed consent was obtained from all participants and their parents. Children were visited at home with their parent (s) present. Trained research team members and data collectors informed children and parents about the procedures and risks of the study and to document the process, obtained written assent and consent from all children and parents, respectively. All phases of the study were approved by the Federal University of Pelotas Ethics Committee, including recruitment, consent/assent procedure, and data collection, protocol, and analysis.

### Participants

Participants were a subsample of the 1993 Pelotas (Brazil) Birth Cohort study (N = 5,249) [15]. At the mean age of 13.3 years, a subsample of the cohort was randomly selected to take part in a detailed study of energy expenditure and physical activity. Those individuals were similar to the remaining cohort members in terms of socioeconomic level and birth weight. Details on the 1993 Pelotas (Brazil) Birth Cohort characteristics are available elsewhere [15]. All data were collected from participants over a ten-day period during the school year. Energy expenditure data was collected over the entire 10-day period. Accelerometry data was collected over four consecutive days of this period, and questionnaire data was collected using a recall of seven days within this period.

### Measurements

Individuals were visited at home where height and weight were measured by trained research staff. Participants were given an Actigraph GT1M accelerometer (Actigraph Corporation, Pensacola FI). Technical details of the device as well as data on its validity are available elsewhere [16]. The Actigraph was placed on the left side of the waist. An instruction sheet was given to participants, containing a brief description of the device, details on how to wear it, and contact information for the researchers. This instruction sheet also included a diary for the devices. Participants were instructed to note if they did not wear the monitor for any period >1 hour during the day. Subjects wore the monitor from Wednesday to Monday and were encouraged to wear it all day, except when showering, bathing, or swimming. Primarily on Monday mornings, fieldworkers visited the participant's home to collect the monitor and the diary, which provided any notes regarding the usage of the device. As a consequence, for most adolescents (>80%), accelerometer data was comprised of four consecutive days (Thursday, Friday, Saturday and Sunday).

The epoch was set to 5 s and data were analyzed using the MAHuffe software (http://www.mrc-epid.cam.ac.uk/Research/PA/Downloads.html). Days with <600 min of registered data, and periods of time above 60 minutes of consecutive zero counts were excluded [17]. For the purposes of this analysis, physical activity variables were expressed as mean counts per minute (cpm), as an indicator of daily average physical activity intensity, and time spent in MVPA, using the Evenson et al. threshold of 2,296 counts [18]. We relied on these cut points because they have higher ability to accurately classify physical activity intensities than other cut points in adolescents [19]. Intensity thresholds were scaled down (division by 12) to accommodate the 5 s epoch setting.

Physical activity was also estimated through a pre-tested and standardized questionnaire. The reliability and concurrent validity of the physical activity questionnaire were tested in a previous study [20]. The reliability was good (rho: 0.62; p<0.001); 73% of the subjects were classified consistently in a seven-day test-retest exercise. The kappa value was 0.58. The concurrent validity of the questionnaire was tested against pedometers; the Spearman correlation coefficient was 0.26 (P = 0.02), and 57% of the subjects were classified consistently as physically inactive in the questionnaire and with pedometers (using a cutoff point of 10,000 steps per day). The questionnaire investigated physical activities related to the mode of transportation to-and-from school, physical activities inside and outside school settings, as well as leisure-time activities. The list of leisure-time activities investigated was created following a pilot study with open-ended questions on the activities practiced more frequently by the adolescents. The final instrument included 15 activities, as well as a blank space for others. For each activity reported, data on weekly frequency and duration were collected. A weekly physical activity score in minutes per week was constructed by multiplying frequency and duration of participation in all types of physical activity.

TEE and body composition were measured using the DLW technique. This dual isotope probe uses the kinetics of two isotopes of water (^2^H_2_O and H_2_^18^O) to quantify the size of the body water pool, and the rate of carbon dioxide production. This technique has been described in detail elsewhere [21], [22]. Briefly, a drink containing both isotopes was administered to the study participant. Urine samples were collected pre-dose, and over the following 10 days. A sample of the dose solution was retained for analysis. Isotopic enrichment was measured using isotope-ratio mass spectrometry. The basic equation for carbon dioxide production rate (rCO_2_) used was as follows:
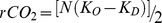



where N is the dilution space of the isotopes, approximately equal to total body water (TBW), and k is the rate constant for either deuterium (D) or 18-oxygen (O) [21].

In this study, the dose solution contained 2.5 g/kg of 10% H_2_^18^O and 0.1 g/kg of 99.9% ^2^H_2_O. The amount of dose consumed was recorded accurate to 0.01 g, by weighing the bottle before and after dosing. Urine samples were analyzed for ^2^H and ^18^O enrichment using isotope-ratio mass spectrometry, using a Gasbench-Delta XP system (Thermofisher Delta Plus and Gas-bench, Bremen, Germany) after equilibration with 2% H_2_ in He for measurement of ^1^H/^2^H and 0.3% CO_2_ in He for measurement of ^18^O/^16^O. All samples were analyzed in duplicate. Dilution spaces and flux rates of the ^2^H and ^18^O tracers were calculated as described previously, allowing calculation of total energy expenditure using established equations [21], [22]. An assumed value of 0.85 was used for respiratory quotient. TEE error was calculated from internal errors on isotopic dilution spaces and flux rates and expressed as a percentage of the final value, as described previously [21], [23].

The dilution space for each isotope was calculated from disappearance curves using the back extrapolation method. The following equation was used to estimate TBW from the two spaces, based on previous studies quantifying the magnitude of overestimation of TBW by N_D_ and N_O_ [24].
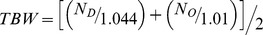


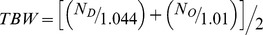



Lean mass was calculated from TBW using recently published values for the hydration of lean tissue [25], using the following equation:
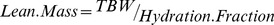



Fat mass was then calculated as the difference between lean mass and weight.

BMR was predicted from weight, height, age using the equations of Schofield, and subtracted from TEE to calculate PAEE [14]. Preliminary analysis showed that dividing PAEE by weight produced an index (PAEE in kcal/kg/d) that was not correlated with weight (rho  = 0.10; p = 0.6). Therefore, this outcome was considered independent of weight.

### Analyses

We initially described the sample using descriptive statistics. We then used Spearman coefficients to evaluate the correlation among variables. We prepared scatter plots with TEE or PAEE on the y-axis and physical activity by accelerometry or questionnaire on the x-axis. We then used linear regression models to examine the contribution of physical activity variability to explain variability in TEE or PAEE. We used the adjusted r^2^ value to express the proportion of the variability explained by each predictor and by the combination of them. Analyses were run in Stata and a significance level of 5% was used in all analyses.

## Results

DLW data from 25 cohort members were analyzed. Data from five participants was incomplete and not used. **Table 1** describes the participants in terms of body composition, energy expenditure, and physical activity. TEE estimated by DLW varied from 1,265 to 4,143 kcal/day. The mean PAEE was 811.2 kcal/day. The N_D_/N_O_ space ratio mean was 1.05 (SD 0.02). Boys were more active than girls by accelerometry (delta  = 15 minutes of MVPA per day; 95%CI 9, 21; p<0.01), but TEE and PAEE did not differ significantly between boys and girls (p = 0.38 and 0.75, respectively). Boys tended to have more fat-free mass (delta  = 2.8 kg; 95%CI −3.1, 8.8; p = 0.33) and less fat mass (delta  = −3.8 kg; 95%CI −9.2, 1.6; p = 0.16) compared to girls, although the differences were not statistically significant.

**Table 1:** Description of the sample in terms of body composition, energy expenditure and physical activity.

Variable	All	Boys (n = 9)	Girls (n = 16)	
	*Mean (SD)*	Mean (SD)	*Mean (SD)*	P
Age (years)	13.0 (0.3)	12.9 (0.3)	13.1 (0.3)	0.35
Weight (kg)	51.5 (13.1)	51.9 (12.2)	51.7 (9.5)	0.97
Height (cm)	159.2 (7.5)	159.5 (10.5)	159.0 (5.6)	0.88
Fat-free mass (kg)	38.2 (6.8)	40.1 (7.6)	37.3 (6.5)	0.33
Fat mass (kg)	14.2 (6.3)	11.8 (6.8)	15.6 (5.8)	0.16
Total energy expenditure (kcal/day)	2541 (688)	2707 (766)	2443 (669)	0.38
Physical activity energy expenditure (kcal/day)	811 (544)	859 (575)	783 (542)	0.75
N_D_/N_O_ space ratio	1.05 (0.02)	1.04 (0.02)	1.05 (0.02)	0.50
Accelerometry (min/day)				
Sedentary	660 (80)	660 (82)	661 (78)	0.97
Light	189 (45)	200 (48)	177 (37)	<0.01
Moderate	63 (27)	69 (27)	58 (25)	<0.01
Vigorous	8 (6)	10 (7)	6 (5)	<0.01
Self-reported physical activity (min/wk)	318 (450)	441 (517)	185 (314)	<0.01

Values are means +/− standard deviations (SD).

In **Table 2** **,** we present Spearman correlation coefficients of TEE and PAEE by DLW with physical activity indicators. TEE and PAEE were inversely correlated to sedentary time, but the correlations were not statistically significant. Light-intensity physical activity was significantly correlated with PAEE from DLW. Similarly, moderate intensity and the combination of moderate and vigorous intensity activities (MVPA) were also significantly correlated with TEE and PAEE. In contrast, vigorous intensity (VPA) was not associated with any of the DLW derived measures, suggesting a smaller contribution of VPA compared with light and moderate-intensity physical activity to TEE and PAEE. Physical activity estimated by questionnaire correlated positively with TEE by DLW (rho  = 0.37; P = 0.04), but not with PAEE (rho  = 0.30; p = 0.15).

**Table 2:** Spearman correlation coefficients between total energy expenditure (TEE) and physical activity energy expenditure (PAEE) by doubly labeled water and physical activity by accelerometry and questionnaire.

	TEE (kcal/day)		PAEE (kcal/day)	
Physical activity	Rho	P	Rho	P
Accelerometry (min/day)				
Sedentary	−0.32	0.12	−0.20	0.34
Light	0.32	0.11	0.43	0.04
Moderate	0.56	**0.004	0.61	<0.01
Vigorous	0.23	0.27	0.37	0.07
Moderate-vigorous	0.57	**0.003	0.62	<0.01
Questionnaire (min/wk)				
Physical activity	0.41	*0.04	0.30	0.15

Spearman's rank coefficients (Rho) are based on kilocalories per day of Total Energy Expenditure (TEE) and Physical Activity Energy Expenditure (PAEE).

**Figure 1:**
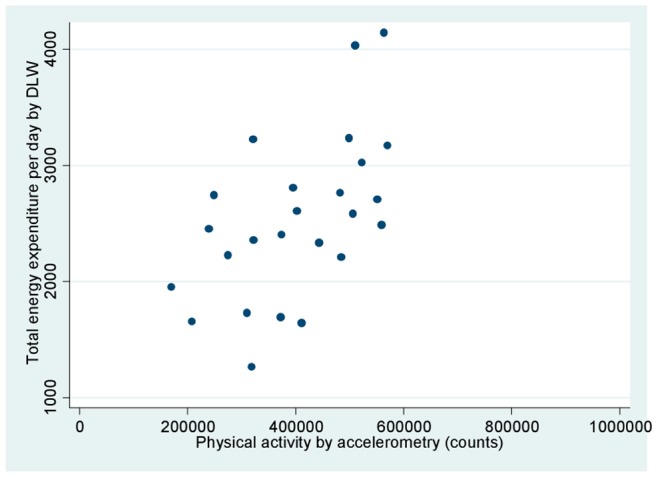
Total energy expenditure (TEE) by doubly labeled water (kcal/day) and accelerometry-based physical activity (counts). The regression equation is TEE  = 1221+ (0.0033 * counts), adjusted r^2^ = 0.31, p-value for accelerometry-based physical activity 0.002.

**Figure 2:**
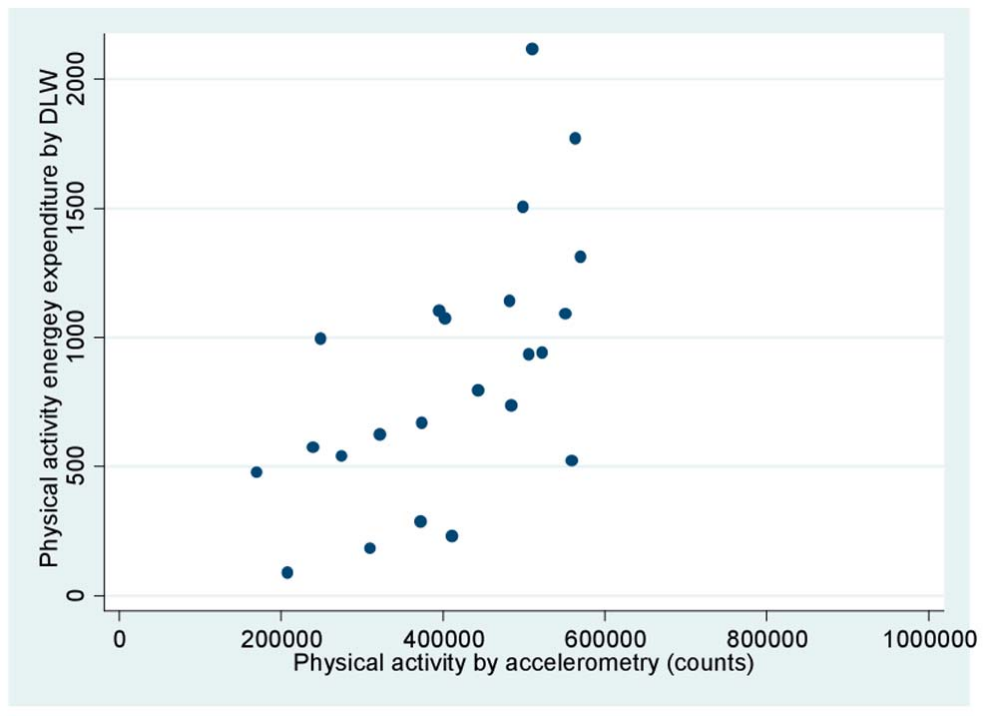
Physical activity energy expenditure (PAEE) by doubly labeled water (kcal/day) and accelerometry-based physical activity (counts). The regression equation is PAEE  = −286+ (0.0027 * counts), adjusted r^2^ = 0.34, p-value for MVPA 0.002.

**Figure 1** plots TEE against physical activity by accelerometry. A significant trend of increasing TEE with increasing MVPA was apparent. Among adolescents classified as active by accelerometry (+60 minutes of MVPA per day), the mean TEE was 2,807 kcal/day, as compared to 1,965 kcal/day among those classified as inactive. In **Figure 2** **,** we present the comparable plot using PAEE instead of TEE. Again, increasing PAEE was significantly related to increasing MVPA, although the slope of the line was different from that for TEE (m = −286 for PAEE compared to m = 1221 for TEE).

**Figure 3:**
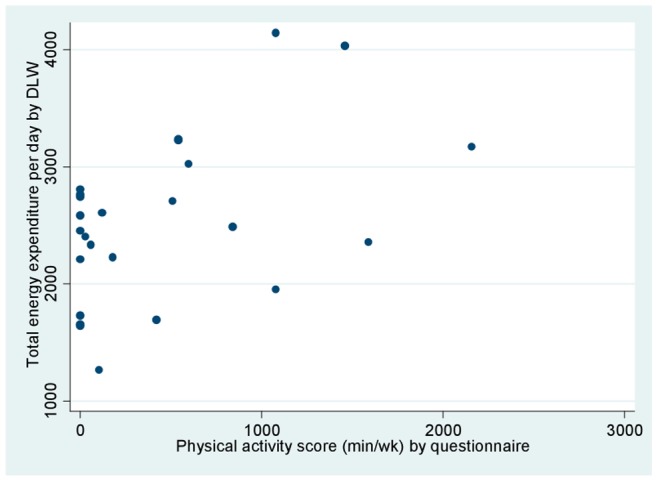
Total energy expenditure (TEE) by doubly labeled water (kcal/day) and minutes per week spent on physical activity by questionnaire. The regression equation is TEE  = 2283+ (0.56 * minutes per week of physical activity), adjusted r^2^ = 0.20, p-value for physical activity 0.02.

**Figure 4:**
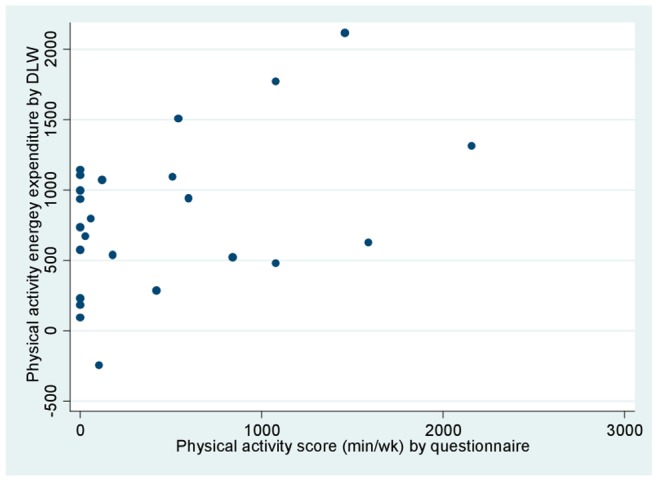
Physical activity energy expenditure (PAEE) by doubly labeled water (kcal/day) and minutes per week spent on physical activity by questionnaire. The regression equation is PAEE  = 637+ (0.39 * minutes per week of physical activity), adjusted r^2^ = 0.16, p-value for physical activity 0.03.

**Figure 3** and **4** plot the questionnaire data against TEE and PAEE by DLW, respectively. Although a general positive relationship was observed, some points distorted the trend, particularly some individuals with zero minutes per week of physical activity by questionnaire and considerably high energy expenditure by DLW. Individuals classified as active by the questionnaire (+300 minutes per week of physical activity) presented an average TEE of 2,910, as compared to 2,245 among those classified as inactive (p<0.01) (Figure 3).

**Table 3** presents regression statistics for the prediction of TEE and PAEE by accelerometry and questionnaire variables and body composition. In a simple linear regression, physical activity by accelerometry (counts) explained 31% of the variability in TEE and 36% of the variability in PAEE (Table 3). An increment of 10 minutes per day in MVPA was equivalent to an increase of 156 kcal/day in TEE. In unadjusted analyses, both fat mass (rho  = 0.56; p<0.01) and fat-free mass (rho  = 0.48; p = 0.01) were correlated with TEE. However, for PAEE the correlation was significant for fat mass (rho  = 0.50; p = 0.01) but not for fat-free mass (rho  = 0.23; p = 0.27). By incorporating fat mass and fat-free mass in the regression model, 58% of the variability in TEE was explained, although the association of TEE with fat mass was not statistically significant. Noteworthy, the coefficient related to accelerometry-based physical activity was only marginally attenuated in the adjusted model; an increment of 10 minutes per day in MVPA was equivalent to an increase of 141 kcal/day in TEE. For PAEE, neither fat mass nor fat-free mass were significant in the model, and the attenuation of the physical activity coefficient was modest.

**Table 3:** Linear regression on the predictors of total energy expenditure (TEE) and physical activity energy expenditure (PAEE) by doubly labeled water and moderate-to-vigorous physical activity (MVPA) by accelerometry.

	MVPA				MVPA + Body Composition			
Dependent Variables	B	SE	p	r^2^	B	SE	p	r^2^
TEE:								
Constant	1361	360	<0.01	0.31	−498	655	0.38	0.58
MVPA (minutes/week)	15.6	4.5	<0.02		14.1	3.7	<0.01	
Fat-free mass (kg)					40.4	16.7	0.03	
Fat mass (kg)					29.6	17.0	0.10	
PAEE:								
Constant	−142	273	0.58	0.36	−801	590	0.19	0.43
MVPA (minutes/week)	12.6	3.4	<0.01		10.8	3.4	<0.01	
Fat-free mass (kg)					11.5	15.1	0.42	
Fat mass (kg)					25.3	15.3	0.11	

Values are presented in linear regression coefficients (B) with standard error (SE).

## Discussion

In the present study, we compared physical activity measured by accelerometry and questionnaire against energy expenditure measured by the DLW method. Our findings show that physical activity continuous scores measured using either accelerometers or questionnaires correlate with energy expenditure measured by DLW in Brazilian adolescents.

The stronger correlation of accelerometry compared to questionnaires with energy expenditure aligns with the current knowledge. In our study, physical activity assessed by accelerometry was a significant predictor of both TEE and PAEE while self-reported physical activity was associated with TEE but not PAEE. Despite questionnaires being the most practical and cost-effective tool to measure physical activity in large-scale studies and the preferred option for physical activity surveillance worldwide, they may incorporate some bias due to relying on self-report, particularly in studies with children [26]. Alternatively, more costly accelerometry techniques have been found to have stronger correlations with energy expenditure than questionnaires, because they provide objective measures of body movement [26].

Accelerometer-based physical activity was strongly associated with TEE, explaining 36% of the variance. There have been three previous studies in children and adolescents that also found positive correlations [3], [4], [7] and two studies that failed to detect any association [5], [6]. Similar to our findings, Hoos et al. reported a positive association between physical activity by accelerometry and TEE by DLW among a group of children aged 7–9 years old (n = 11) [4]. Ekelund and colleagues also associated TEE and PAEE with accelerometry in a sample of 26, 9-year olds from Denmark; they also found an independent association of TEE with fat-free mass [3]. Montgomery and colleagues found that although energy expenditure was not influenced by engagement in MVPA, it was influenced by time spent sedentary and in light-intensity activities in a younger group of 104 Scottish children (mean age  = 5.4 years) [7]. In contrast, Krishnaveni and colleagues found no significant association between accelerometer-based MVPA and TEE among 58 children 8–9 years old (n = 58), similarly to the findings reported by Johnson and colleagues in a study with 31 children aged, on average, 8.3 years [6]. It is important to bear in mind that in the Krishnaveni study [6] had little variability in activity counts within the sample, and that the Johnson study [5] had a heterogeneous sample in terms of age and did not express movement in raw units.

The differences in the magnitude of agreement between studies may be due to different accelerometer devices being used and the age of participants being studied [6]. MVPA levels measured by accelerometry explained approximately ∼1/3 of the variability in TEE and PAEE. These findings align with literature that has found that agreement between accelerometry-based physical activity and energy expenditure is dependent on the activity level of the population; whereby, the agreement between these two measures stands to be higher when studying populations of lower physical activity levels than more active groups [9], [10]. Thus, in this current study, agreement will potentially be higher because of studying older, adolescent children when physical activity levels start to decline, especially in girls. The fact that self-reported physical activity was significantly related to TEE, but not PAEE, might be explained by greater error on PAEE due to combining two different raw estimates.

By also incorporating fat mass and lean mass, almost 60% of the variability in TEE was explained, thus suggesting that both activity levels and fat mass and fat-free mass are at least of the same importance in determining TEE. Ekelund and colleagues concluded that adjustment for fat-free mass removed the confounding effect of sex on PAEE in children and adolescents [14]. In our study, there was no sex difference in TEE. Nevertheless, our study supports the notion that fat mass and fat-free mass factors could affect accelerometer-based energy expenditure calculations, and this is important to take into account.

Questionnaire-based physical activity was also associated with TEE, but not with PAEE. Similar to our study, Corder et al. compared PAEE by DLW against four separate physical activity questionnaires among children aged 4–5, 12–13, and 16–17 years old and found that PAEE could be correctly assessed and ranked at the group-level but not the individual level by two of the four questionnaires used [13].

There were several strengths of this study. The study included the comparison of both accelerometers and physical activity questionnaires against the gold standard for measuring energy expenditure, DLW, in a group of Brazilian adolescents. Further, adjustments were made for body composition including fat mass and fat-free mass. Studies of this type, particularly using DLW, of youth in low- to middle-income countries are limited; therefore, this study adds significantly to the body of literature in this area of research. This study also uses a comparison of DLW and accelerometry using commonly used protocols. Previously, similar studies have used a ten-day period to collected accelerometry data to match the protocol for DLW data collection. While this accurately assesses their agreement, participants in free living accelerometry studies typically wear accelerometers for fewer days. We therefore decided to test the agreement using real-world protocols.

Limitations include the small sample size, due in large part to the high cost of the 18-oyxgen isotope when used in individuals of large body size, and the fact that due to time constraints, BMR, used in the estimation of PAEE, was predicted using equations rather than measured directly through indirect calorimetry. Finally, we have no data on diet induced energy expenditure and were unable to estimate body composition using other techniques, such as the four-compartment model. Another issue to be discussed is the fact that nine individuals had a physical activity score through self-report of 0 minutes per week. It means they do not regularly use active modes of transportation to school and did not engage in any leisure-time activity lasting for 10 or more consecutive minutes in the previous week. Obviously, these subjects did spend some energy; part of the discrepancy between self-report and other methods is explained exactly by the fact that only bouts of 10 consecutive minutes or more are typically reported in questionnaires.

Because associations have been shown to vary by population and age group, our study is also limited by the lack of power to test sex-interactions. Another possible limitation is that some of the activities performed by these adolescents, such as carrying weight, are not well captured by accelerometry. However, we also had a list of activities practiced by the adolescents, and the most frequently reported ones were soccer (boys) and walking (girls) – both are well captured by accelerometry. Finally, some of the discrepancy between methods could be due to non-activity related energy expenditure, particularly due to growth. This is particularly relevant at this age range. Further studies that assess these variations are needed on this topic, particularly in low- and middle-income countries where 84% of the world's population live and the highest burden of non-communicable diseases (NCDs) take place [27].

## Conclusion

Objectively measured physical activity significantly contributes to the explained variance in both TEE and PAEE in Brazilian youth. Independently, body composition also explains variance in TEE, and should ideally be taken into account when using accelerometry to predict energy expenditure values.
